# Microevolution of *Aedes aegypti*

**DOI:** 10.1371/journal.pone.0137851

**Published:** 2015-09-11

**Authors:** Caroline Louise, Paloma Oliveira Vidal, Lincoln Suesdek

**Affiliations:** 1 Laboratório Parasitologia, Instituto Butantan, São Paulo, SP, Brasil; 2 Programa de Pós-Graduação em Medicina Tropical, Instituto de Medicina Tropical, Universidade de São Paulo, São Paulo, SP, Brasil; 3 Programa de Pós-Graduação em Biologia da Relação Patógeno-Hospedeiro, Instituto de Ciências Biomédicas, Universidade de São Paulo, São Paulo, SP, Brasil; Universidade Federal do Rio de Janeiro, BRAZIL

## Abstract

Scientific research into the epidemiology of dengue frequently focuses on the microevolution and dispersion of the mosquito *Aedes aegypti*. One of the world’s largest urban agglomerations infested by *Ae*. *aegypti* is the Brazilian megalopolis of Sao Paulo, where >26,900 cases of dengue were reported until June 2015. Unfortunately, the dynamics of the genetic variability of *Ae*. *aegypti* in the Sao Paulo area have not been well studied. To reduce this knowledge gap, we assessed the morphogenetic variability of a population of *Ae*. *aegypti* from a densely urbanised neighbourhood of Sao Paulo. We tested if allelic patterns could vary over a short term and if wing shape could be a predictor of the genetic variation. Over a period of 14 months, we examined the variation of genetic (microsatellites loci) and morphological (wing geometry) markers in *Ae*. *aegypti*. Polymorphisms were detected, as revealed by the variability of 20 microsatellite loci (115 alleles combined; overall F_st_ = 0.0358) and 18 wing landmarks (quantitative estimator Q_st_ = 0.4732). These levels of polymorphism are higher than typically expected to an exotic species. Allelic frequencies of the loci changed over time and temporal variation in the wing shape was even more pronounced, permitting high reclassification levels of chronological samples. In spite of the fact that both markers underwent temporal variation, no correlation was detected between their dynamics. We concluded that microevolution was detected despite the short observational period, but the intensities of change of the markers were discrepant. Wing shape failed from predicting allelic temporal variation. Possibly, natural selection (Q_st_>F_st_) or variance of expressivity of wing phenotype are involved in this discrepancy. Other possibly influential factors on microevolution of *Ae*. *aegypti* are worth searching. Additionally, the implications of the rapid evolution and high polymorphism of this mosquito vector on the efficacy of control methods have yet to be investigated.

## Introduction

*Aedes* (Stegomyia) *aegypti* Linnaeus 1762 is a widely distributed mosquito vector of dengue, the most important arboviral disease in humans. The prevention and control of the dengue virus currently depend on controlling its mosquito vector. Different methods have been proposed for dengue vector control, but many of these methods are limited by the microevolution of mosquitoes.

In this context, the demography, dispersion, and evolution of these insects within urban areas have been frequently investigated. Additionally, these biological issues are of importance to professionals engaged in vector control and surveillance initiatives but are far from being satisfactorily understood. Dispersal is preferably estimated by population genetics indicators, such as gene flow, migration and genetic variability, given the difficulty and low reproducibility of mark-release-recapture methods for small mosquitoes [1–3].

Genotypic markers such as Single Nucleotide Polymorphisms (SNPs) and microsatellite loci have been largely used to investigate the microevolution of *Ae*. *aegypti* throughout the world [4–8], but the changes in the rates of allelic frequencies are variable and unique to each study case. Phenotypes may also help to describe microevolution, as in the example of wing geometry. Wing shape in insects is heritable and evolutionarily informative [9, 10]. Wing morphometrics alone were sensitive enough to detect microevolution and geographical variation in species of *Aedes* [10–12].

Despite the usefulness of microsatellite loci and wing shape, these markers have been seldom combined in a single study of biological patterns of *Aedes* spp. [12], and as far as we know, they have not been associated in microevolutionary investigations. After the publication of Vidal and Suesdek [12] and Vidal et al. [10], we formulated the following related hypotheses: 1) allelic profiles of *Ae*. *aegypti* changes over short evolutionary time periods; 2) genetic microevolution can be assessed based on wing phenotype.

To test these hypotheses, we evaluated the temporal morphogenetic variations of a single population of *Ae*. *aegypti* over 14 months using microsatellites and wing geometry as biological markers. The chosen population came from "Subprefeitura Butanta", a small, homogeneous and densely urbanised neighbourhood of Sao Paulo City (Brazil). Globally, this city is one of the largest urban agglomerations infested by *Ae*. *aegypti*, with approximately 11,000,000 people distributed over an area of 1,523 sq. km. There were 31,101 cases of dengue reported in this City in 2014, and more than 26,900 cases were reported from January to June 2015 [13].

## Materials and Methods

### Specimen collection

**Fig 1:**
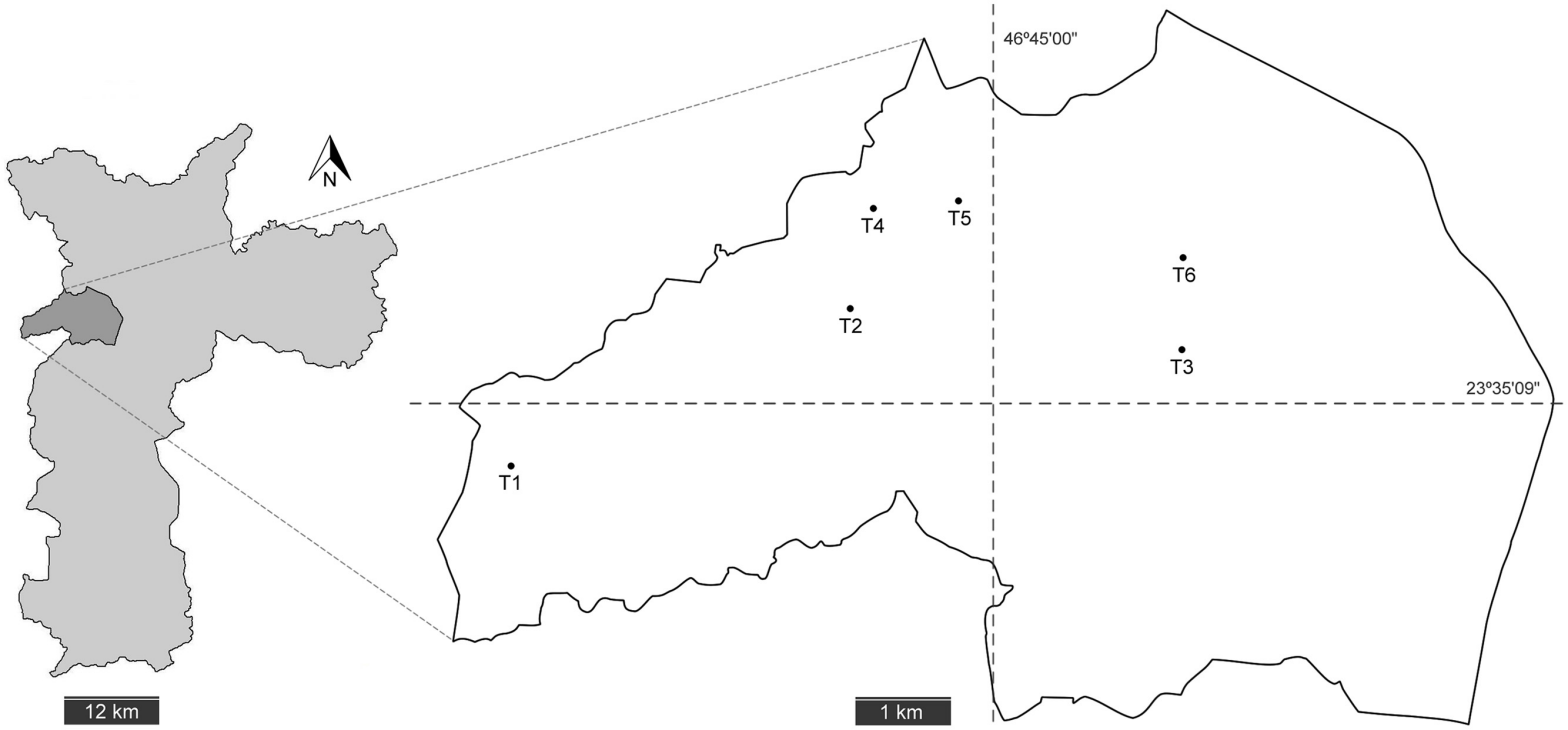
Sampling locations of *Aedes aegypti*. Left: outline map of Sao Paulo City showing in detail the political boundaries of neighbourhood “Subprefeitura Butanta”. Right: Magnified outline of “Subprefeitura Butanta” depicting the exact location of each egg trap.

Eggs, pupae and larvae of *Ae*. *aegypti* were collected in “Subprefeitura Butanta”, a relatively small, geopolitically delimited neighbourhood comprising approximately 3% of the Sao Paulo municipality, which is homogeneously and densely urbanised (visualisation available at https://www.google.com.br/maps). Specimens were obtained from six traps placed throughout the neighbourhood (see map in Fig 1). Each trap consisted of grouped water containers comprising ~1 L volume. SUVIS is a governmental entity which helped us in the field collecting and it has a permanent authorization to collect in the private properties where traps T1-5 were located. Trap T6 was inside the private property of MSc. Vivian Petersen, who authorized the procedure. The traps were distributed in such a small area in order to maximize the chances of sampling only one populational deme: traps T1-5 permitted sampling only a single neighbourhood, whereas trap T6, even more exclusive, permitted sampling only one backyard (see Table 1 for details).

**Table 1:** Sampling information—*Aedes aegypti*.

Trap	Geographical coordinates	Habitat	Chronological samples
T1	23°35'31.55"S, 46°48'12.98"W	Car wrecks	AUT11
T2	23°34'38.21"S, 46°45'57.30"W	Used tires	AUT11
T3	23°34'52.02"S, 46°43'47.42"W	Car wrecks	AUT11
T4	23°34'2.71"S, 46°45'47.90"W	Used tires	WIN
T5	23°34'0.22"S, 46°45'15.37"W	Used tires	WIN
T6	23°34'19.58"S, 46°43'47.35"W	Egg trap	SPR, SUM and AUT12

Fourteen monthly collections were conducted between April 2011 and May 2012, and to equalise sample sizes, the samples were pooled into five chronological samples according to their respective climatic seasons: autumn 2011 (AUT11), winter 2011 (WIN), spring 2011 (SPR), summer 2011/12 (SUM) and autumn 2012 (AUT12) (For more details, see Table 1). Immature mosquitoes were maintained in the laboratory with a natural photoperiod and standard conditions of temperature and humidity (25±1°C; 80±10%). The emerging adult mosquitoes were identified at a species level and stored at -80°C until they were analysed. In total, 150 adult females were analysed, 30 from each of the five chronological samples.

### Genetic analysis

Genomic DNA was extracted with the DNeasy Blood and Tissue Kit (Qiagen, USA) or standard DNA isolation [12] (149 in total; DNA from one individual SPR sample was misplaced). Genetic polymorphisms of 20 microsatellite loci were assessed: AED19, C2A8, T3A7 (described by Huber et al. [14]); A10, B07, B19 (described by Chambers et al. [15]); AC1, AC2, AC4, AC7, AG1, AG2, AG3, AG4, AG5, AG7, CT2 (described by Slotman et al. [16]); A1, B1 and B3 (described by Brown et al. [17]). For the loci described by Huber et al. [14] and Chambers et al. [15], each PCR reaction consisted of 1X PCR buffer (Thermo Scientific, Lithuania), 2.0 mM MgCl_2_, 0.4 mM of each dNTP, 10 pmol of each primer, 0.20 U of Taq polymerase and 2 μL of DNA, with a final volume of 25 μL. DNA was amplified in a Mastercycler personal thermal cycler (Eppendorf, Germany). For the loci described by Slotman et al. [16] and Brown et al. [17], amplification was performed using Platinum Multiplex PCR Master Mix (Life Technologies, USA).

The thermal PCR conditions for all loci were according to Huber et al. [14], Chambers et al. [15], Slotman et al. [16] and Brown et al. [17]. The annealing temperature for the loci were as follows: 56°C for the AED19 locus, 64°C for the T3A7 locus, 60°C for the A10, B07 and B19 loci and 55°C for all the other loci. The 5’ end of the forward or reverse primer was labelled with a fluorescent dye (either HEX, NED, FAM, VIC or PET) (Bioneer, USA and Life Technologies, USA) appropriate for the ABI 3730 or ABI 3500 automated sequencer (Applied Biosystems, USA).

The PCR products were genotyped using GeneMarker V2.2 software package [18]. The presence and frequencies of null alleles were assessed using Microchecker V2.2.3 [19]. For each chronological sample, parameters of genetic variability were assessed using Genepop V4.2 software [20]. Linkage disequilibrium within pairs of loci was investigated using Genepop V4.2 software. Deviations from Hardy-Weinberg equilibrium (HWE) were tested using Genepop V4.2 software. Significance levels for multiple testing were corrected using the Bonferroni procedure [21]. Allelic richness was calculated using HP-Rare software [22].

To assess the genetic stability over time, several analyses were done. Differentiation among chronological samples was examined by Wright’s F-statistics (F_st_) estimated as described by Weir and Cockerham [23] and Nei’s genetic distance (D) [24], F_st_ and D were computed using Genetix V4.05 [25]. A UPGMA tree was constructed using the PHYLIP package [26] and edited in FigTree V1.4.0 [27]. A factorial correspondence analysis (FCA) was computed using Genetix V4.05. This analysis permits one to visualize latent factors that describe the majority of the variation in the multilocus genotypes.

We also tested the hypothesis that any chronological sample was a genetically distinct population. We then submitted the allele frequencies to a Bayesian model-based clustering test using STRUCTURE V2.3.4 [28] without assuming a priori the number of sampling occasions. To infer the most likely number of population units (K), we calculated the Delta-K value [29], which is based on the second-order rate of change of the likelihood function between successive values of K. We used four independent runs (K = 1 to 10 for all the traps and K = 1 to 6 for the trap T6) with a burn-in value of 500,000 iterations and 500,000 replications.

### Wing shape analysis

**Fig 2:**
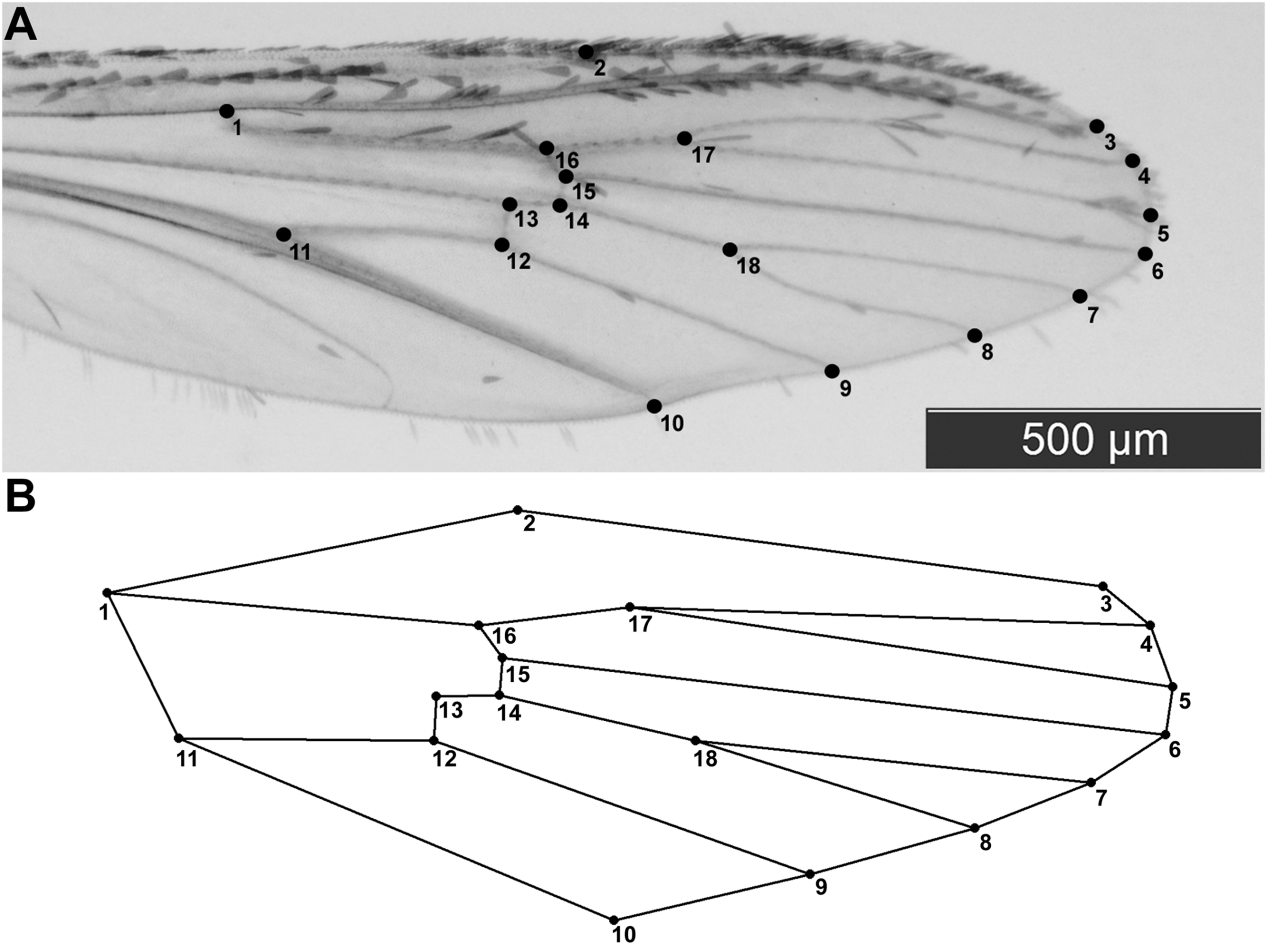
(A) Wing of Aedes aegypti (female) depicting the 18 landmarks chosen. (B) Geometric diagram linking all landmarks.

The right wings of all 150 females were detached, mounted on slides-coverslip and photographed according to Vidal et al. [10]. On each wing image, we digitised 18 landmarks using TpsDig V1.40 software [30] (Fig 2). Slides and their associated images were respectively deposited in Instituto Butantan and WingBank (http://www.wingbank.com.br).

Wing size was estimated by calculating the “centroid size” vector [31]. To assess wing shape, the positional landmark data were submitted to generalised Procrustes superimposition (scaling, rotation and translation), as previously described by Vidal et al. [10]. Covariation of shape variables was assessed by analysis of principal components (PCs). Softwares: TpsUtil V1.46 [32], TpsRelw V1.49 [33] and MorphoJ V1.05 [34].

We estimated the allometric influence of wing size on wing shape through multiple regression analysis of the Procrustes coordinates vs. centroid size. We tested the statistical significance of allometry by means of nonparametric permutation tests with 10,000 iterations using MorphoJ V1.05 software. To geometrically describe wings independently from size variation, allometric effect was removed using multivariate regression.

Chronological samples of *Ae*. *aegypti* were compared regarding wing size and shape in order to evaluate the morphological variation during the observational interim (14 months). Univariate parametric statistic tests were used to compare centroid sizes: ANOVA with *Tukey* post-hoc test implemented using InStat [35].

Discriminant analysis was used to explore the degree of wing shape dissimilarity among individuals in a morphospace of canonical variables (CVs; MorphoJ V1.05 software). To better visualise the shape disparity among the samples, thin-plate splines were constructed based on the regression of CV1 scores on shape coordinates (TpsRegr V1.31 [36]). Discriminant analysis also allowed us to calculate the Mahalanobis distances among samples. To further examine the phenetic relationships among the chronological samples, Mahalanobis distances were used to construct UPGMA phenograms using the PHYLIP package [26], which were edited in FigTree V1.4.0 [27].

Quantitative estimator of intra/inter group differentiation (Q_st_) and metric disparity among samples were estimated and statistically compared by nonparametric permutation tests (2,000 iterations each) using COV software [37]. The dissimilarity between consecutive chronological samples was estimated by cross-validated reclassification tests (MorphoJ V1.05 software).

Morphological diversity was estimated by the “amount of dispersion” of the individuals in the morphospace of PCs. Theoretically, the amount of dispersion of individuals (of a single set) in the morphospace of PCs is proportional to the morphological variability of that population set. In the present case, the amount of dispersion was estimated using the procedure developed by one of us (L.S.) in [38] and was herein modified as follows. The positional (Cartesian) coordinates of plots in the morphospace of PCs (each one corresponding to a single mosquito individual) were digitised using TpsDig software (similarly to which was done to wing landmarks). The centroid size vector of a set of individuals (a chronological sample) in the morphospace was estimated in TpsRelw software. Following that, the value of this vector was used as the estimator of morphological diversity of each chronological sample.

### Correlation between the two markers

In order to confront molecular and morphological data, a Pearson´s correlation analysis was done (α = 0.05) between phenetic and genetic distances (i.e., distances between chronological samples). We also compared between F_st_ scores and morphological Q_st_ scores. Based on the FCA plot, we calculated an index of “genetic diversity” using a similar method to that used to estimate the “morphological diversity” (see wing shape analysis). These genetic and morphological diversities were then compared using linear correlation analysis (α = 0.05).

## Results

### Genetics

#### Description of patterns

All 149 individuals of the five chronological samples were successfully genotyped using 20 microsatellite loci. All loci were polymorphic and comprised in total 115 alleles (mean, 5.75 per locus), which were distributed as follows: 2 (AED19), 4 (C2A8), 7 (T3A7), 7 (A10), 13 (B07), 8 (B19), 5 (AC1), 3 (AC2), 2 (AC4), 6 (AC7), 5 (AG1), 18 (AG2), 5 (AG3), 4 (AG4), 5 (AG5), 7 (AG7), 3 (CT2), 5 (A1), 2 (B2) and 4 (B3). The following unique alleles were detected in AUT11: allele 229 (locus A10, frequency 1.6%); allele 179 (B07, 3.3%); 168 (B19, 3.3%); 133 (AC7, 1.6%); 121 and 133 (AG2, 3.3% and 1.6%); 149 (AG7, 1.6%); 176 (CT2, 5%), WIN: 177 (B07, 3.3%); 207 (B19, 3.4%); 131 (AG2, 1.6%); 154 (B3, 1.6%) and SUM: 151 (AG2, 1.6%) (see S1 Table for more details). The most and least polymorphic chronological samples were, respectively, WIN (100 alleles) and SPR (84 alleles). Estimates of allelic richness for each chronological sample ranged from 4.87 (WIN) to 4.15 (SPR).

In general, genotype distributions were compatible with our premises of neutrality and representative sampling. Linkage disequilibrium was detected in only 29 of 950 (3%) comparisons, and most loci (13 out of 20) were in HWE.

Only some departures from equilibria were significant. The pair of loci AG1-AG3 was in linkage in all chronological samples. The B07, B19, AG1, AG2 and AG4 loci occasionally presented heterozygote deficits (S2 Table). The T3A7 and C2A8 loci were not in HWE (heterozygote deficits) and presented high levels of null alleles (S3 Table). The results of genetic diversity based on FCA analysis are shown in Table 2.

**Table 2:** Scores of genetic diversity and morphological diversity of chronological samples.

Sample	AUT11	WIN	SPR	SUM	AUT12
**Genetic diversity**	1.78	1.14	1.11	1.28	1.30
**Morphological diversity**	0.81	0.75	0.77	1.03	0.84

#### Variation over time

The analyses of genetic fixation (F_st_), genetic distance (D), factorial correspondence analysis (FCA) and clustering (Bayesian model) based on allele frequencies indicated that genetic structure changed over time. Some private alleles were found but all of them appeared in low frequencies. Considering that high levels of null alleles and linkage of loci could bias our interpretation, we performed the analysis with and without C2A8, T3A7 and AG1 loci but the results were quite similar (not shown). The AG1 locus was discarded from the presented analysis (F_st_, D, FCA and Bayesian) because it appeared in linkage not only with AG3 locus but also with other 6 loci.

**Fig 3:**
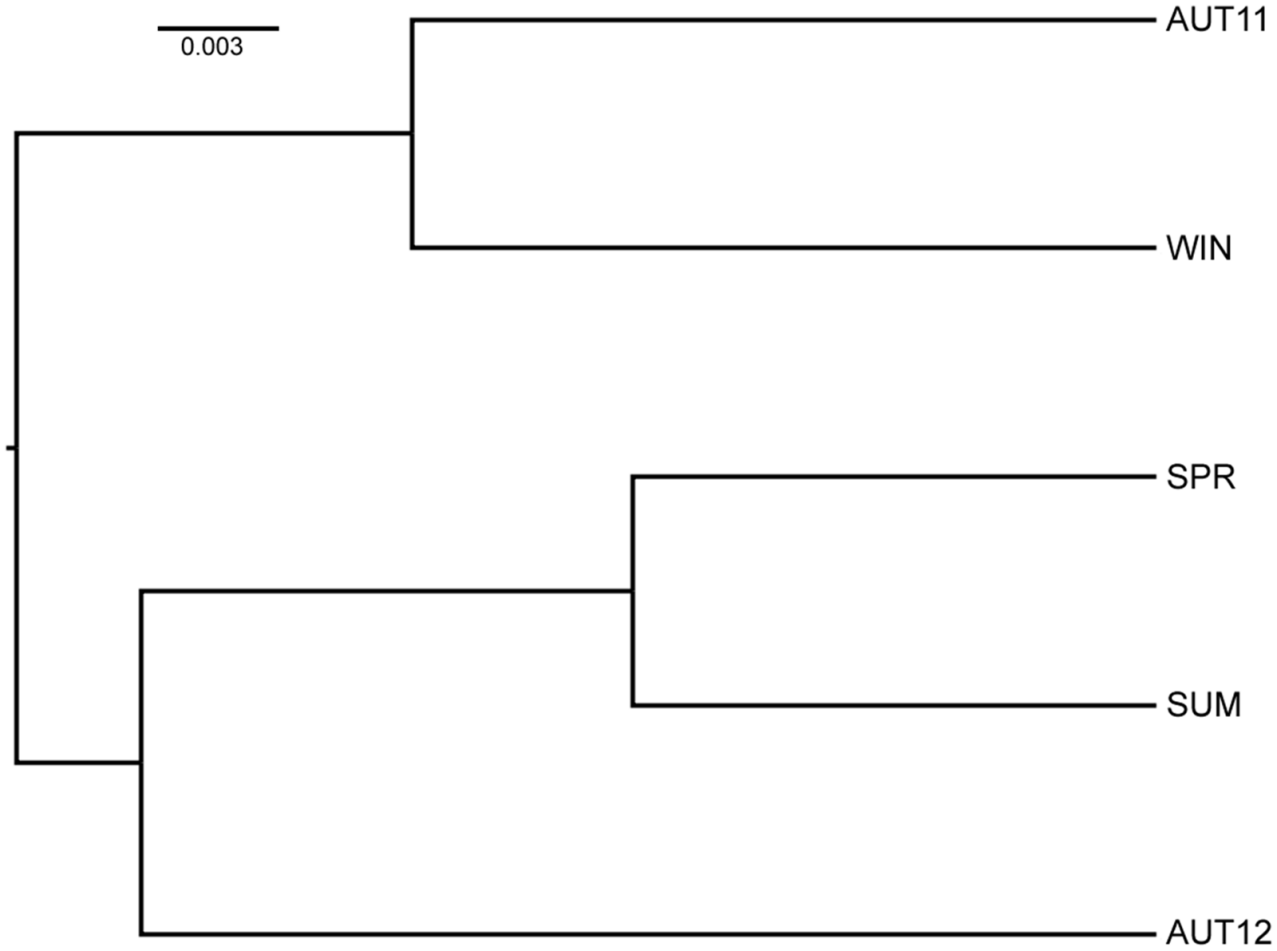
UPGMA dendrogram using Nei’s genetic distance between chronological samples.

**Fig 4:**
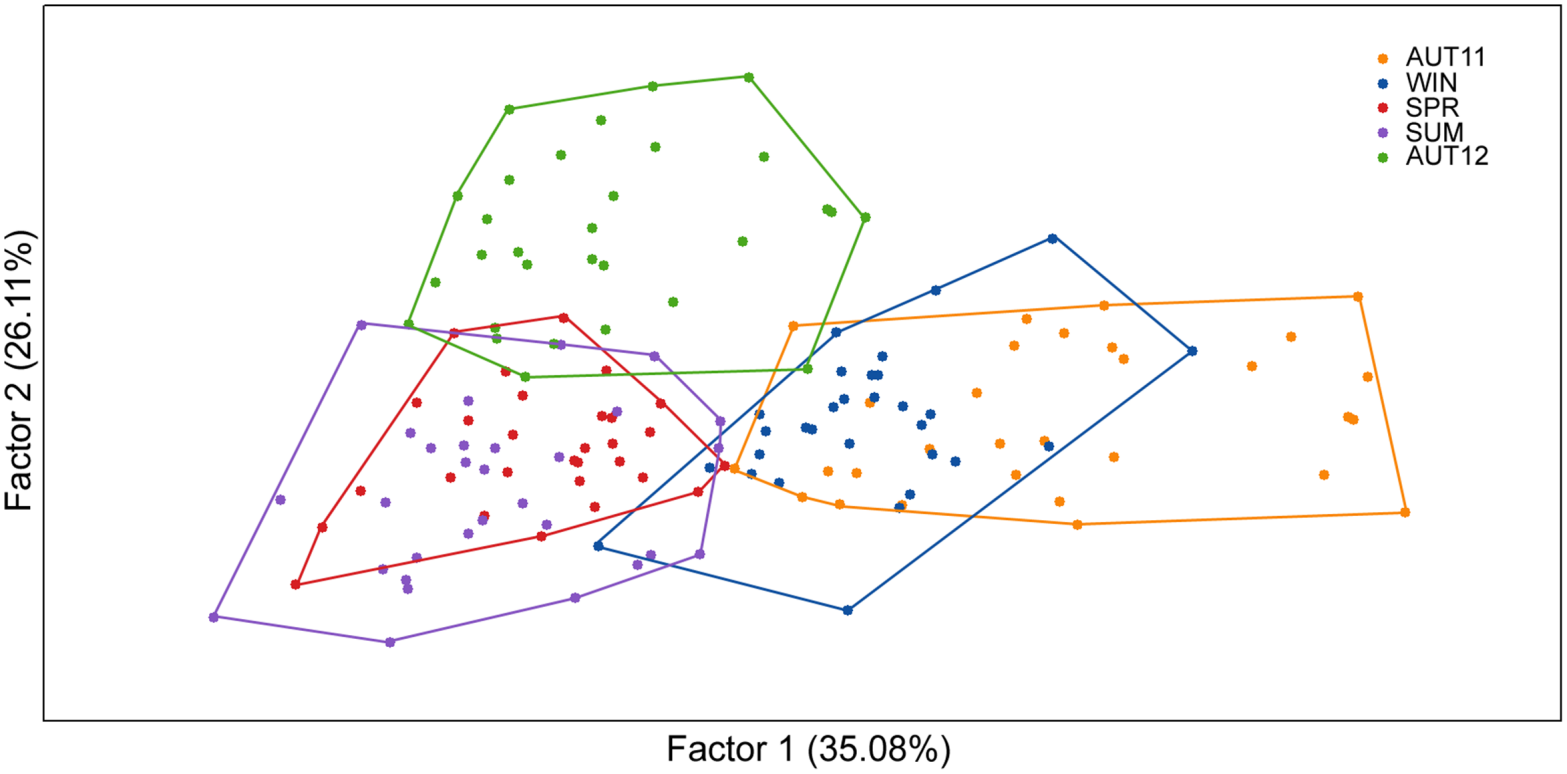
Factorial correspondence analysis of allelic temporal variation. Each polygon represents the multilocus genetic variation of each chronological sample. Between brackets, the relative contribution of each factor (accounted for 61.19% of the total variation).

The overall F_st_ value was low but significant (F_st_ = 0.0370; p<0.05). Pairwise D values calculated over all loci between samples ranged from 0.027 to 0.063 and revealed slight genetic differentiation. The UPGMA dendrogram based on the genetic distance matrix (Fig 3) revealed two major clusters (AUT11, WIN) and ((SPR, SUM) AUT12)). Taken together, these results indicate that the genetic distance between chronological samples was not strictly related to chronological proximity. Accordingly, factorial correspondence analysis indicated temporal genetic differentiation. Variation was not constant during the time, as only some samples overlapped (Fig 4).

**Fig 5:**
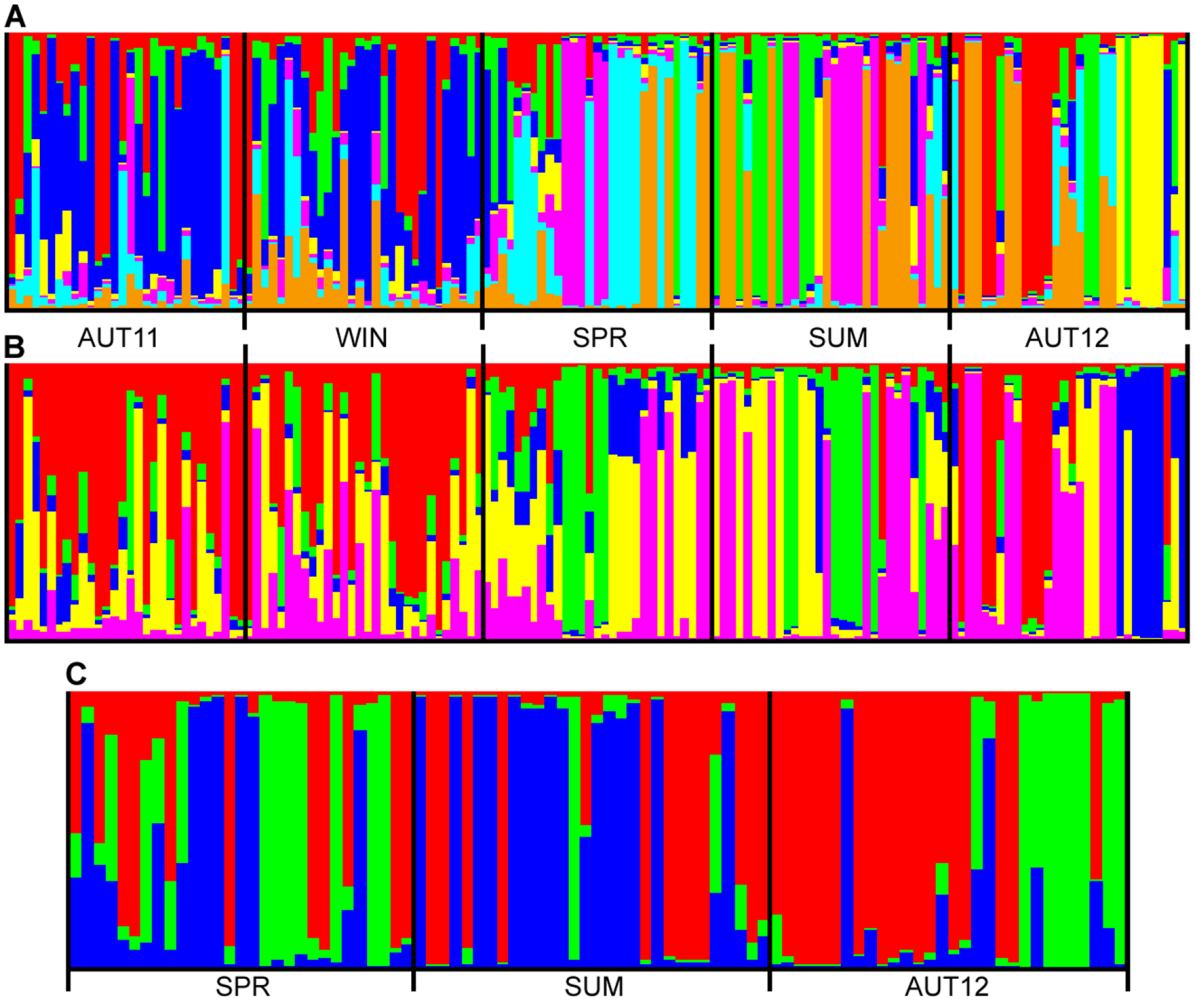
Graphical presentation of the Bayesian model-based clustering analysis. Each individual is represented by a vertical bar. (A) k = 7, the best value of K for all mosquitoes of the chronological samples. (B) k = 5, arbitrarily chosen to match the number of chronological samples. (C) k = 3, the best value of K for all mosquitoes from trap T6.

According to the Bayesian model-based clustering test, the most probable k was k = 7 and no population structure was evidenced. We them assumed k = 5 to match the number of chronological samples, and we noted distinction of the samples from trap T6 (single backyard, SPR + SUM + AUT12), as shown in Fig 5A and 5B. The hypothesis of each chronological sample be a genetically-distinct population was not supported.

In order to evaluate the morpho-genetic syntopic variation (in a single trap location), we also did a sub-analysis with only the samples from trap T6. In this case, the most likely k was equal to 3, which is in accordance to the three seasons sampled in trap T6 (Fig 5C). Thus, an incipient distinction among SPR, SUM and AUT12 was noted. In comparison to the 5-sample analysis, F_st_ and morphological Q_st_, were lower in the sub-analysis (0.0321 and 0.3312, respectively).

### Morphology

#### Wing shape

**Fig 6:**
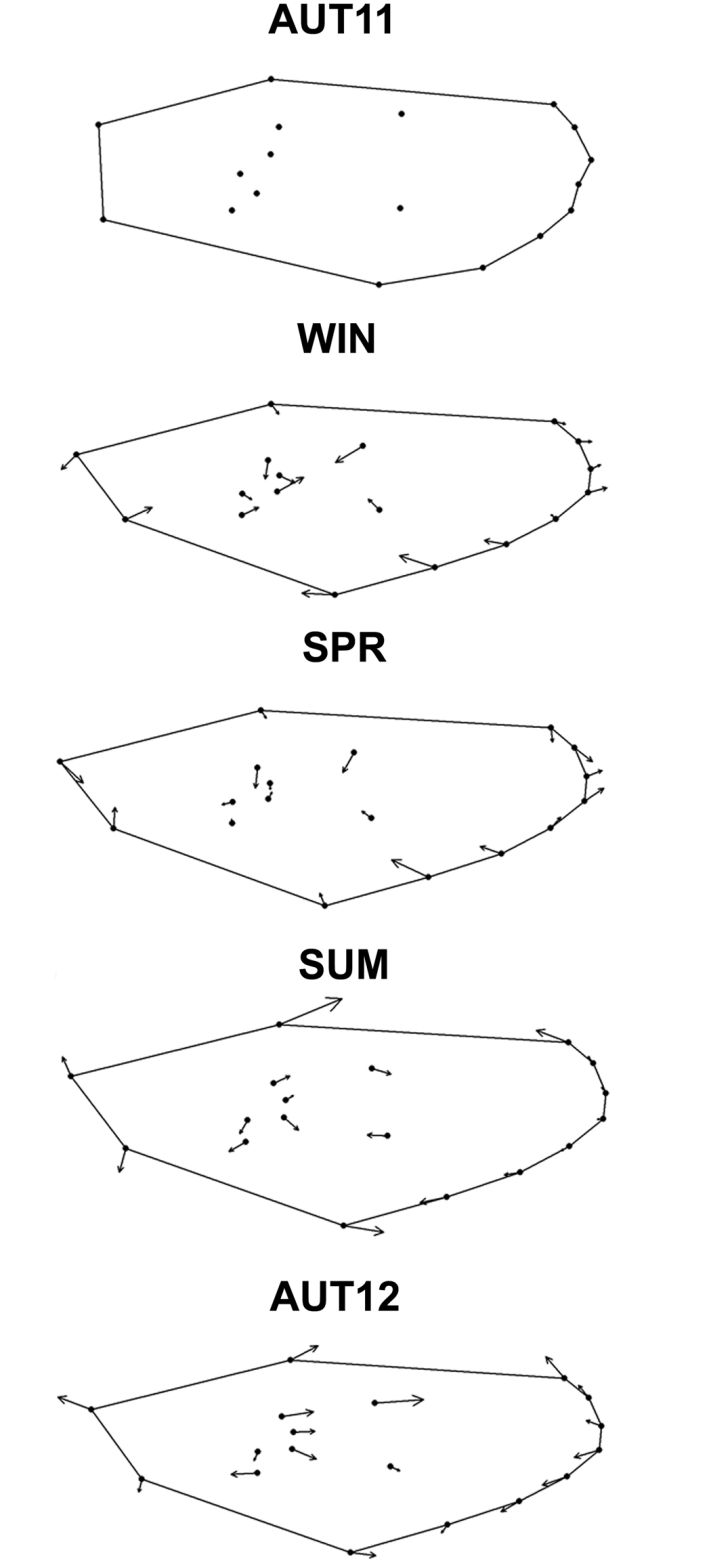
Wing shape temporal variations represented by thin-plate splines. Only the 18 landmarks, the wing outline and deformation vectors are shown. In each chronological sample depicted variations are relative to the previous sample. Deformations were magnified 10X to facilitate visualization.

**Fig 7:**
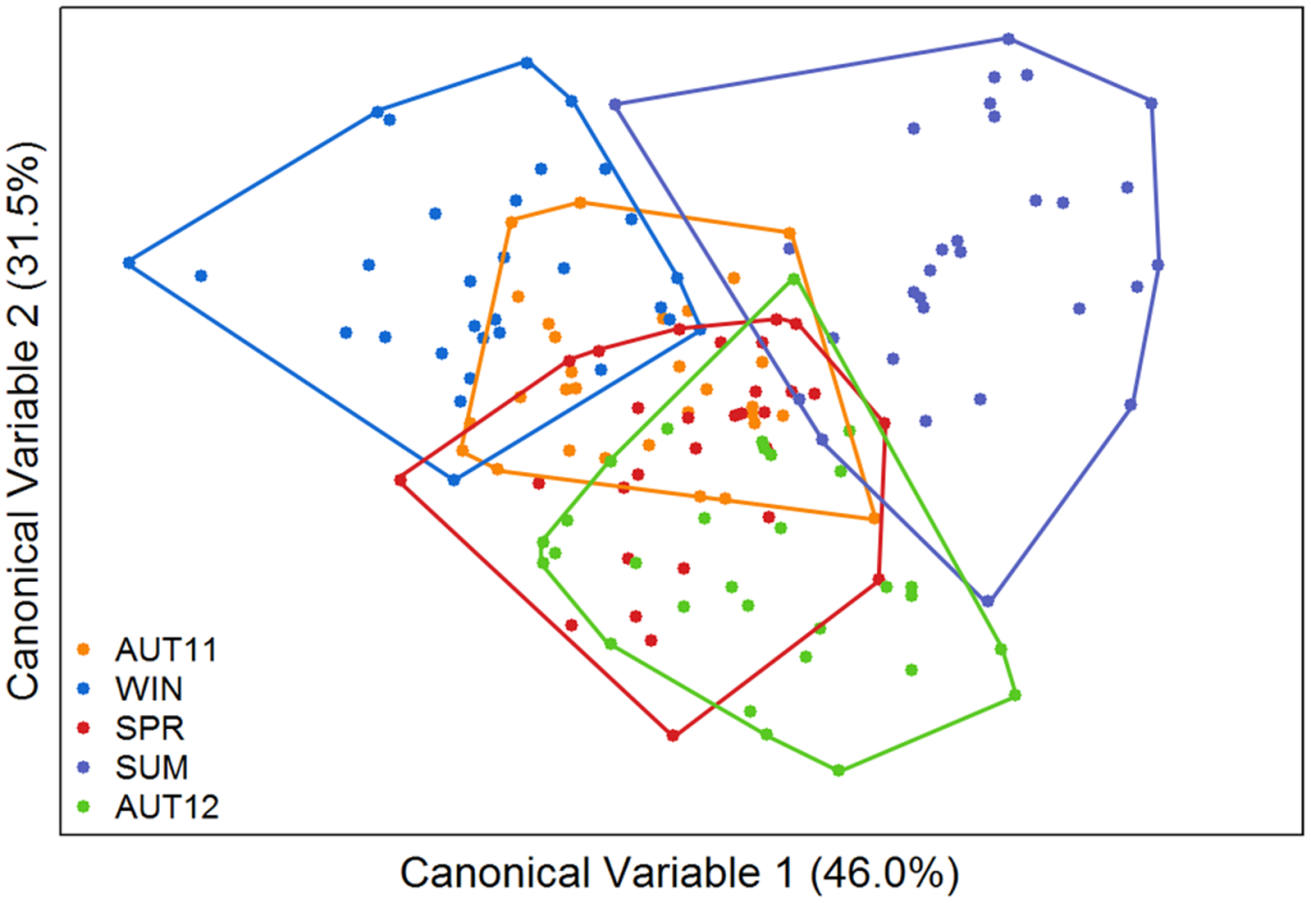
Morphological space of canonical variables (CVs) 1 and 2 yielded by discriminant analysis of wing principal components. Each polygon represents a chronological sample. Relative discriminant power of each variable is between brackets (accounted for 77.5% of the total variation).

The mean shape temporal variation can be visualised in the thin-plate-spline series shown in Fig 6. After the removal of allometry (which significantly accounted for 1.9%; p = 0.0052), discriminant analysis revealed shape differentiation among chronological samples in the morphospace of canonical variables 1 and 2 (Fig 7). The differentiation degrees were unequal, and SUM was the most distinct, whereas SPR-AUT12 was the most similar pair of samples. Additionally, canonical variable 3 (13.2% of the total variation) indicated a distinction between AUT11 and SPR (not shown). The results of morphological diversity based on PCs are shown in Table 2.

**Fig 8:**
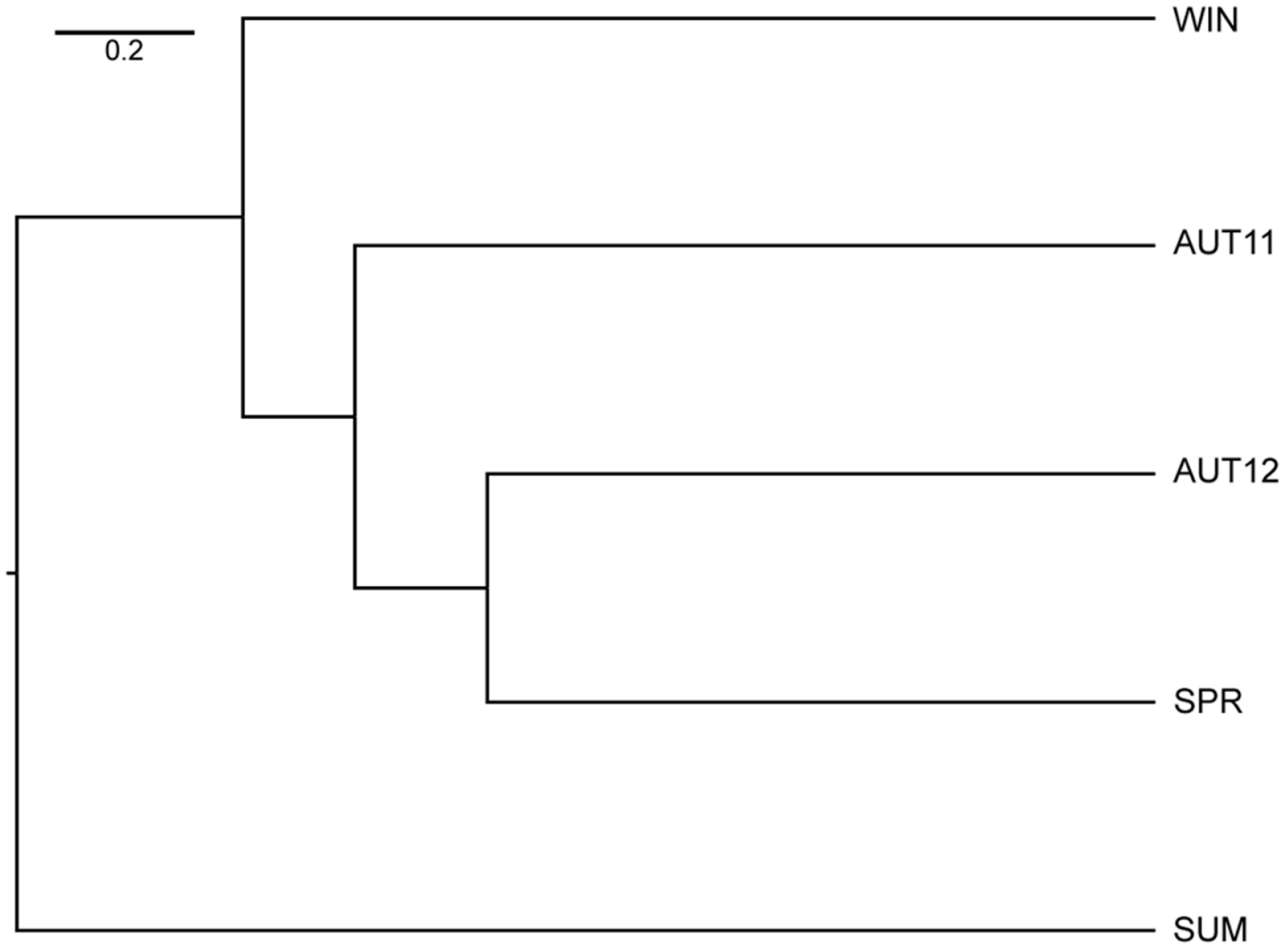
UPGMA phenograms using the Mahalanobis distance between chronological samples.

The phenogram of chronological samples based on Mahalanobis distances (Fig 8) corroborated the view that SUM was the most distinct sample. SPR and AUT12 were the most similar pair, despite not being from chronologically consecutive seasons. All pairwise comparisons among chronological samples exhibited significant metric disparity (p<0.0001; permutation test). Accuracy scores of the cross-validated reclassification tests based on Mahalanobis distances ranged from 60–83.3% (Table 3). The overall morphological quantitative estimator (Q_st_) was 0.4732.

**Table 3:** Scores of reclassification pairwise tests after validation (Only consecutive pairs were tested for reclassification).

COMPARISON		
AUT11 (70%)	X	WIN (80%)
WIN (60%)	X	SPR (80%)
SPR (70%)	X	SUM (70%)
SUM (76.6%)	X	AUT12 (83.3%)

#### Wing size

Statistical analysis (ANOVA; α<0.05) of the consecutive chronological comparisons revealed that wing size increased from AUT11 to WIN, remained stable during SPR, decreased to the lowest level in SUM and reached its highest point in AUT12 (see S1 Fig for more details). Variance of size was high in all samples.

## Discussion

### Genetic variability

The population genetics parameters (HWE, low linkage disequilibrium, few null alleles) indicated that our genetic markers are representative and our geographically-restricted collecting has successfully sampled within a single deme. Variability at the microsatellite loci was high, and the rate of change was not homogeneous. Allelic frequency profiles (S1 Table) indicated that temporal genetic variation was rather quantitative than qualitative. Additionally, the F_st_ value indicated that the genetic compositions of chronological samples were not identical. However, the rate at which allelic frequencies changed pointed to a slight differentiation, but was not high enough to result in recognisable “populations” according to the Bayesian cluster analysis. In general, the observed genetic variation is indicative of microevolution. Remarkably, the samples from a single backyard (trap T6), which is the most syntopic sampling, is a clear demonstration of the how microevolution may occur during a short period (three consecutive seasons).

It is not possible to do a complete comparison between our genetic results and the data available in the literature because, as far as we know, there are not other studies which comprise all the 20 loci set employed here. We can say that, in general, the population was highly polymorphic, because the observed allelic diversity and richness are seldom found in a single population of *Ae*. *aegypti*. Even in the colder season (WIN), the genetic variability was high, what is unexpected to an exotic species (*Ae*. *aegypti* is not native from the Neotropics), as introduced populations usually undergo genetic drift and founder effect. This may reflect a rich genetic patrimony and may imply adaptation of this species to new environments, which is a limiting factor to control efforts.

The loci used here exhibited, on average, similar polymorphism levels as those reported for *Ae*. *aegypti* populations from other locations across the world [14, 15, 17, 39–42]. For example, we found 13 alleles for the B07 locus, whereas populations from Trinidad had 11 alleles [15] and populations from Manaus had 6 alleles [40]. At the AG2 locus, we found 18 alleles, whereas populations from Manaus had 3 alleles [40], and populations around the world had 29 alleles [42].

Allelic variation in *Ae*. *aegypti* over time has been occasionally studied. Ordinarily, allelic frequencies of microsatellites do not change greatly in a few years. For example, another population of *Ae*. *aegypti* from Sao Paulo State (Brazil) exhibited dimorphic SNPs that remained stable for 6 years [7]. Genetic stability between climatic seasons was previously reported in Australia [4] and between two years in the southern islands of the USA [6]. Nevertheless, there are cases in which rapid changes occur. In northern Brazil, *Ae*. *aegypti* exhibited allelic variation during the transition of dry/wet seasons, possibly driven by a bottleneck effect [40] and Olanratmanee et al. [8] detected seasonal allelic fluctuations within two years in Thailand. An example of a closely related species that also evolved rapidly (just a few years) is *Aedes japonicus*, which arguably occurred as a result of the merging of two genetically distinct lineages [43].

### Morphological variation over time

The wing shape/size patterns of *Ae*. *aegypti* from the Brazilian neighbourhood “Subprefeitura Butanta” changed over the study interval. This is the first investigative study of the temporal variation of the wing characters of *Ae*. *aegypti*.

Regarding wing shape, the significant metric disparity among all samples and the high scores of cross-validated reclassifications showed that wing shape evolved and diverged during the 14 months interval, a remarkably short time period. The levels of temporal divergence were so high as to be compatible with partial population substitution, which, however, was not supported by the microsatellite results. The most similar case in the literature is a study of the congeneric *Aedes albopictus* [10], the wing shape of which changed yearly for four years.

Wing shape polymorphisms were observed, denoted by the high value of the Q_st_ indicator (0.4732), and the level of polymorphism remained high in each chronological sample during the study interval, as indicated by the morphological diversity estimates (Table 2). No clear association between climatic and morphological variations was detected. Even in the summer when the hot and humid climates typically leads to demographic explosions of mosquitoes [44], morphological diversity was only slightly higher than the average. Nevertheless, the SUM sample was the most divergent regarding the shape (Fig 7), but there is no theoretical or empirical support to establish a relationship between this pattern and the climatic variables.

Concerning wing size, we cannot interpret much from the evolutionary point of view because this character is considered labile [9]. Accordingly, we analysed the wing shape after removing the allometric effect. We observed that the mean sizes might be distinct, even between equivalent climatic seasons (AUT11-AUT12; S1 Fig), suggesting that microenvironmental and/or genetic factors have a greater influence upon this trait.

Apparently, the velocity of the microevolution of *Ae*. *aegypti* depends not only on its genetic patrimony but also on its ecological context. We do not know which local aspects of Sao Paulo are the primary influences on the rapid evolution of *Ae*. *aegypti* in Subprefeitura Butanta, but some factors are worth investigating. For example, the urban landscape is dense and continuous, and there are numerous houses with backyards with water containers; the daily weather is highly variable, and the neighbourhood (as well as the whole city) is a nationally important centre of trade and people travelling. Even when a single trap was analysed (trap T6), both morphological and genetic variations occurred, indicating that microevolution can be detected over just a few syntopic generations.

### Interpreting the two markers combined

Biological and instrumental limitations prevent wings and microsatellites from being completely mutually congruent. The analysis and interpretation of microsatellites are not equivalent to that of wing morphometrics because of the distinct nature of these markers. Microsatellites are diploid, presumably neutral and independent multiallelic loci [45], whereas wing shape is a complex phenotype determined by a quantitative polygenic heritage [9, 46–48].

In fact, results of the two types of markers were discrepant and wings patterns changed faster. The oscillation of morphological diversity was uncorrelated to the genetic diversity. This analysis was not shown, but it is noticeable by comparison between Figs 4 and 7. Consistently, no correlation was found between morphological and genetic distances (r^2^ = 0.0845; r = -0.2908; p = 0.4151). The observed Q_st_ value was several times higher than the F_st_ value, indicating that morphological variability was lower intra-sample than inter-sample. This pattern is typically interpreted as if natural selection is acting on wing shape [9, 49].

Similarly, a previous paper of ours [12] analysed four geographical populations of *Ae*. *aegypti* from Sao Paulo City (including older samples from Subprefeitura Butanta) and observed Q_st_>F_st_; however, wings morphology appeared to be more stable than microsatellite allelic patterns. To understand this partial contradiction between that study [12] and the present study, we re-analysed those data using another comparative approach: metric disparity between the samples based on Mahalanobis distances. Revisitation of those data showed that those four populations were actually significantly distinct, which is in accordance with our interpretation of the current study case. Notwithstanding, one must consider that the two studies are not directly comparable because the previous study [12] was atemporal and comprised a larger geographical scale.

It would be reasonable to think that wing shape is under selective pressure because wings are important to flight and mating behaviour [50]. However, most of the selected landmarks do not appear to play a definitive role in wing aerodynamics or ability to producing courtship sounds.

An alternative explanation for the observed rapid variation of the wing characters is that quantitative polygenic traits (as supposedly occurs to insect wings [9]) may exhibit more phenotypes than the number of genotypes (differential expressivity). In such a case, the observations of phenotypes could lead to super-estimations of genetic variability and microevolutionary rates. Combining this possibility with the evidence that the chronological samples actually comprise a single population unit, we cannot conclude that the temporal variation in the wings was due to immigrants from abroad. Unfortunately, these questions will only be answered when all genes involved with wing determination have been identified and their heritage has been described.

Although the wing does not permit us to predict fine parameters of population genetics, it may be a semi-quantitative indicator of variation and variability. The fact that wing shape is sensitive to short temporal units ascribes discriminant power to these markers. However, the absence of correlation with neutral genetic markers indicate that wing shape may not be suitable to assess microevolution. This conclusion is different from that about the usefulness of wing geometry to macroevolutionary approaches. For instance, wing shape has been increasingly proven to be adequate to diagnose species and cryptic taxa [9, 11, 51–55].

In this specific ecological context, we assume that the dispersal of *Ae*. *aegypti* individuals is not broad because Subprefeitura Butanta has numerous breeding sites. This assumption is in accordance with previous observations that dispersal is restricted when breeding sites are more available [8, 56].

Microevolution has relevant implications in epidemiology. Rapid allelic frequency changes may involve non-neutral characters and occasionally affect the susceptibility of mosquitoes to insecticides and chemical repellents. The successful insertion of transgenic or benign mosquito lineages may also be influenced by microevolution.

The development of these processes is multi-causal, and a central factor is the ecological context. A comprehensive understanding of the ecological context requires careful studies that consider biotic/abiotic local peculiarities. We propose that not only the static biological patterns but also the evolutionary dynamics of *Ae*. *aegypti* should be further investigated and taken into consideration when redesigning vector control and surveillance protocols.

## Supporting Information

S1 Fig Descriptive boxplots of wing centroid sizes (in mm). Significant distinctions: AUT11 X AUT12 p<0.01, WIN X SUM p<0.01, SPR X SUM p<0.01 and SUM X AUT12 p<0.001.
(TIF)

S1 Table Allele frequencies in 20 loci microsatellites. (XLSX)

S2 Table Genetic variability at loci microsatellite on chronological samples. N, sample size (number of mosquitoes); AR, allelic richness; H_o_, observed heterozygosity; H_e_, expected heterozygosity; F_is_, inbreeding coefficient. In bold, significant p-values (α<0.05) following Bonferroni correction (corrected α<0.0005) rejecting Hardy—Weinberg equilibrium.—not applicable.
(XLSX)

S3 Table Null allele estimates per locus. In bold, significant p-values (p>0.01).—not applicable.
(XLSX)
