# Supplementary figures and images for: Microevolution of Aedes aegypti
Source: PLoS One. 2015 Sep 11;10(9):e0137851. doi: 10.1371/journal.pone.0137851 (PMC4567268; doi:10.1371/journal.pone.0137851)

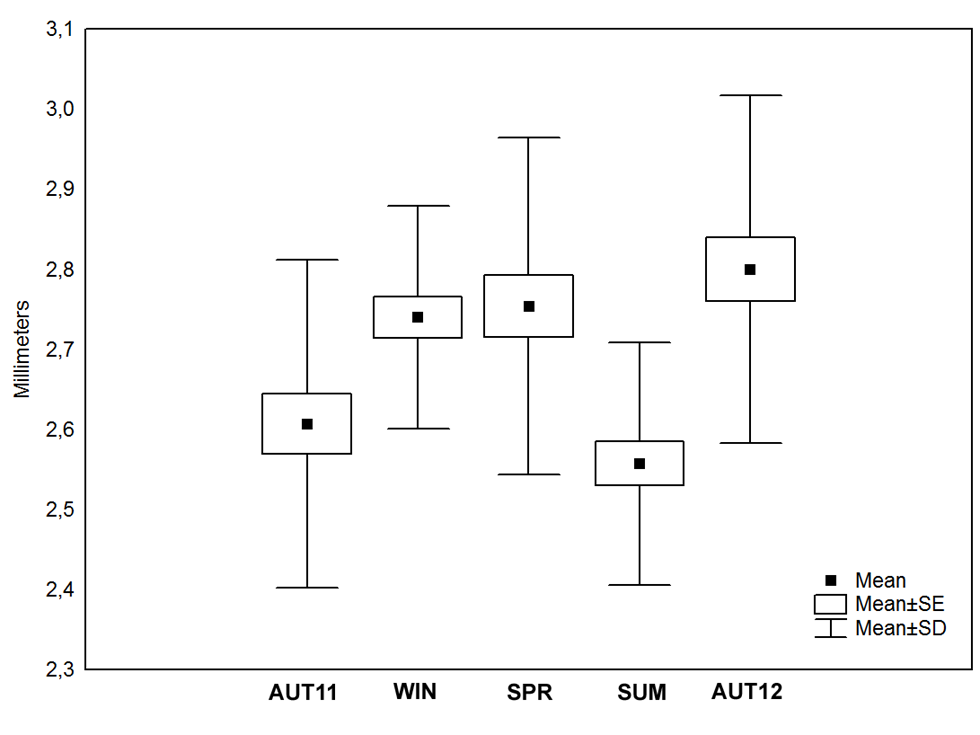

Supplement: S1 Fig — Significant distinctions: AUT11 X AUT12 p<0.01, WIN X SUM p<0.01, SPR X SUM p<0.01 and SUM X AUT12 p<0.001. (TIF) [file pone.0137851.s001.tif]
